# Trauma induced coagulopathy and fibrinogen levels: why do we need to measure them, and what are the supplementation strategies?

**DOI:** 10.5935/2965-2774.20230132-en

**Published:** 2023

**Authors:** Felicio Savioli

**Affiliations:** 1 Department of Critical Care Medicine, Hospital Sírio-Libanês - São Paulo, Brazil

## INTRODUCTION

Fibrinogen is a large glycoprotein produced in the liver. With a normal plasma concentration of 1.5 - 3.5g/L, fibrinogen is the most abundant blood coagulation factor. The final stage of blood clot formation is the conversion of soluble fibrinogen to insoluble fibrin, leading to a stable clot.^(1)^ In cases of severe bleeding, fibrinogen reaches critically low plasma concentrations at an earlier stage than other coagulation factors.^(2)^ Fibrinogen also binds to glycoprotein IIb/IIIa receptors on the platelet membrane, promoting platelet aggregation and clot stabilization.^(3)^ The importance of fibrinogen in clot firmness can be better illustrated by an analogy to a wall, as proposed by Lang and von Depka.^(4)^ If we consider the platelets as bricks and fibrinogen as cement, a balanced ratio allows us to build a stable wall or a stable clot. On the other hand, if the number of bricks is reduced (thrombocytopenia) and the amount of cement is increased (hyperfibrinogenemia), the wall will not break, and the clot will be stable due to the higher levels of fibrinogen. However, if there are bricks but no cement (hypofibrinogenemia), the risk of wall collapse is high or, analogously, the risk of bleeding is high as well.

## TRAUMA-INDUCED COAGULOPATHY

Trauma is one of the 10 leading causes of death and disability in the world and is the leading cause of death in the young population. Moreover, hemorrhage is responsible for at least 40% of deaths after trauma.^(5)^ In a Brazilian hospital, a retrospective study including 467 trauma patients was conducted. Sixty percent of deaths occurred in 24 hours. Of these patients, 18% died from hemorrhage.^(6)^ Trauma-induced coagulopathy (TIC) is a consequence of tissue hypoperfusion and tissue injury. The coagulation system balance changes rapidly during injury and resuscitation, and consequently, the TIC phenotype can quickly change over time from primarily an anticoagulant to a procoagulant state within hours to days if the patient survives. Several processes, including dysfunction of natural anticoagulants, platelet dysfunction, hyperfibrinolysis and fibrinogen consumption, have been identified as primary components of TICs.^(7)^ In major trauma, key contributors to low levels of fibrinogen include hemodilution (due to fluid resuscitation), blood loss, consumption in clot formation at the wound sites, hypothermia, and acidosis.^(8)^ In addition, upon admission, hypofibrinogenemia in major trauma is independently associated with an increase in injury severity and shock and is a predictor of mortality.^(9)^ Currently, the sixth edition of the European guidelines on the management of major trauma following coagulopathy recommends fibrinogen supplementation for patients with bleeding and fibrinogen levels below 1.5g/L.^(10)^

## FIBRINOGEN SUPPLEMENTATION STRATEGIES

There are three possible sources of fibrinogen: fresh frozen plasma (FFP), cryoprecipitate and fibrinogen concentrate (FC).

Fresh frozen plasma contains a variable amount of fibrinogen and other coagulation factors. A prospective cohort study found no consistent correction of clot function or increases in procoagulant factor concentrations following FFP transfusion during the acute phase of ongoing bleeding.^(11)^ Furthermore, the RETIC trial showed that FFP was insufficient to correct hypofibrinogenemia or significantly improve the clot strength versus the fibrinogen concentrate in adult trauma patients. In addition, the study was terminated early for futility and safety reasons because of the high proportion of patients in the FFP group who required rescue therapy compared with those in the FC group.^(12)^ Additionally, the panel of experts from the European Guidelines of Trauma do not recommend fibrinogen supplementation with FFP to correct hypofibrinogenemia if cryoprecipitate or FC are available. According to the guidelines,^(10)^ the evidence was grade 1C.

Cryoprecipitate is derived from FFP and consists of factor VIII, fibrinogen, von Willebrand factor, factor XIII, fibronectin, and other plasma proteins, such as alpha-2 antiplasmin, which decreases fibrinolysis. Variability of the clotting factor levels in blood donors means that the fibrinogen concentration in cryoprecipitate varies as well.^(13)^ CRYOSTAT-1 was a feasibility study for a multicenter, randomized controlled trial evaluating the effects of early administration of high-dose cryoprecipitate in adult trauma patients. This 1-year study recruited 43 patients, with all completing a subsequent 3-month follow-up visit. The authors concluded that early cryoprecipitate therapy maintained acceptable blood fibrinogen levels during active bleeding, with a signal for reduced mortality in the treatment arm of the study.^(14)^ CRYOSTAT-1 facilitated CRYOSTAT-2 trial development. In the CRYOSTAT-2 trial, the effect of early cryoprecipitate (within 90 minutes of admission) compared to standard blood transfusion therapy on 1,605 bleeding trauma patients from 26 major trauma centers has been tested. Data analysis is now underway. This study will provide the answer as to whether early cryoprecipitate transfused for major trauma improves mortality.^(15)^ Currently, the most widespread form of fibrinogen replacement in the UK, Canada and Australia is through the delivery of cryoprecipitate, contrasting with most of Europe, where FC is the preferred method of replacement.^(16)^

In most European countries, FC is the main product for replacing fibrinogen because of its increased viral safety profile, standardized concentration of fibrinogen, lower risk of TRALI and TACO, benefits of room temperature and faster administration.^(17)^

The FiiRST trial described a pilot feasibility study to evaluate the effect on clinical and laboratory outcomes and complications of early infusion of FC in trauma cases. Fifty hypotensive adult patients requiring blood transfusion were randomly assigned to receive either 6g of FC or placebo. The authors observed that early infusion of FC was feasible and led to an increased plasma fibrinogen concentration during trauma resuscitation.^(18)^

Recently, the FlinTIC trial^(19)^ assessed whether FC in the prehospital setting could improve blood clot stability in TICs. Patients were allocated to receive either FC or placebo at the trauma scene or during transportation. A dose of 3g of the study drug was given to patients with bodyweights of 30 to 60kg, 4.5g to patients with bodyweights of 60 to 90kg and 6g to patients with bodyweights of 90 to 120kg. The median between-group difference in the change in FIBTEM MCF was 5mm. The authors concluded that early FC administration was feasible for prehospital trauma care. Additionally, they mentioned that FC supplementation protects against early fibrinogen depletion and promotes rapid blood clot initiation and clot stability.

## CONCLUSION

Hypofibrinogenemia is an important cause of bleeding in trauma patients. The treatment remains fibrinogen replacement with cryoprecipitate or fibrinogen concentrate. However, there is no evidence showing that fibrinogen concentrate is superior to cryoprecipitate in cases of trauma-induced coagulopathy. Larger multicenter trials are necessary to elucidate the best fibrinogen supplementation strategy in trauma patients.
